# Adenosine promotes vascular barrier function in hyperoxic lung injury

**DOI:** 10.14814/phy2.12155

**Published:** 2014-09-28

**Authors:** Jonathan Davies, Harry Karmouty‐Quintana, Thuy T. Le, Ning‐Yuan Chen, Tingting Weng, Fayong Luo, Jose Molina, Bhagavatula Moorthy, Michael R. Blackburn

**Affiliations:** 1 Division of Neonatal‐Perinatal Medicine, Department of Pediatrics, Baylor College of Medicine, Houston, Texas; 2 Department of Biochemistry and Molecular Biology, The University of Texas – Houston Medical School, Houston, Texas

**Keywords:** Adenosine, hyperoxic lung injury, vascular barrier function

## Abstract

Hyperoxic lung injury is characterized by cellular damage from high oxygen concentrations that lead to an inflammatory response in the lung with cellular infiltration and pulmonary edema. Adenosine is a signaling molecule that is generated extracellularly by CD73 in response to injury. Extracellular adenosine signals through cell surface receptors and has been found to be elevated and plays a protective role in acute injury situations. In particular, ADORA2B activation is protective in acute lung injury. However, little is known about the role of adenosine signaling in hyperoxic lung injury. We hypothesized that hyperoxia‐induced lung injury leads to CD73‐mediated increases in extracellular adenosine, which is protective through ADORA2B signaling pathways. To test this hypothesis, we exposed C57BL6, *CD73*^*−/−*^*,* and *Adora2B*^*−/−*^ mice to 95% oxygen or room air and examined markers of pulmonary inflammation, edema, and monitored lung histology. Hyperoxic exposure caused pulmonary inflammation and edema in association with elevations in lung adenosine levels. Loss of CD73‐mediated extracellular adenosine production exacerbated pulmonary edema without affecting inflammatory cell counts. Furthermore, loss of the ADORA2B had similar results with worsening of pulmonary edema following hyperoxia exposure without affecting inflammatory cell infiltration. This loss of barrier function correlated with a decrease in occludin in pulmonary vasculature in *CD73*^*−/−*^ and *Adora2B*^*−/−*^ mice following hyperoxia exposure. These results demonstrate that exposure to a hyperoxic environment causes lung injury associated with an increase in adenosine concentration, and elevated adenosine levels protect vascular barrier function in hyperoxic lung injury through the ADORA2B‐dependent regulation of occludin.

## Abstract (precis)

e12155

Hyperoxic lung injury is characterized by cellular damage from high oxygen concentrations that lead to an inflammatory response in the lung with cellular infiltration and pulmonary edema, and extracellular adenosine has been found to be elevated and plays a protective role in acute injury situations; however, little is known about the role of adenosine signaling in hyperoxic lung injury. We hypothesized that hyperoxia‐induced lung injury leads to CD73‐mediated increases in extracellular adenosine, which is protective through ADORA2B signaling pathways. Our results demonstrate that exposure to a hyperoxic environment causes lung injury associated with an increase in adenosine concentration, and elevated adenosine levels protect vascular barrier function in hyperoxic lung injury through the ADORA2B‐dependent regulation of occludin.

## Introduction

Oxygen was first reported to be used in the acute care of respiratory disease by George Holzapple in 1885 (Heffner 2013). Today, oxygen supplementation is used commonly to treat a wide range of patients from premature neonates to the elderly and a wide range of diseases including pneumonia, COPD, acute lung injury, and acute respiratory distress syndrome (Cordingley and Keogh 2002; Stoller et al. 2010). However, high levels of oxygen exposure are known to cause pulmonary inflammation and injury (Kallet and Matthay 2013) and can contribute to the development of chronic respiratory disease such as Bronchopulmonary Dysplasia (Northway et al. 1967; Chess et al. 2006). High levels of oxygen produce reactive oxygen species (ROS) including superoxide anion (O_2_^−^), hydrogen peroxide (H_2_O_2_), hydroxyl radical (OH^−^), and peroxynitrite anion (ONOO^−^) that overwhelm antioxidants to cause cellular damage and cell death (Kallet and Matthay 2013). This in turn leads to recruitment of immune cells to the lung and loss of pulmonary barrier function, producing the inflammation and pulmonary edema that are characteristics of hyperoxic lung injury.

Adenosine is a nucleoside signaling molecule that has been recognized to play an important role in the regulation of inflammation following acute lung injury (Eckle et al. 2008a,b; Schingnitz et al. 2010; Karmouty‐Quintana et al. 2013). The concentration of adenosine is normally low in the extracellular compartment, but following acute lung injury, extracellular adenosine concentrations rapidly increase (Volmer et al. 2006; Eckle et al. 2007). Adenosine is generated extracellularly in response to cellular injury from adenosine triphosphate (ATP). Cellular injury produces an efflux of ATP that is subsequently dephosphorylated by the membrane bound enzymes CD39 and CD73 to form adenosine. Experiments using *CD39‐* and *CD73*‐deficient mice resulted in decreased extracellular adenosine production following acute injury and demonstrated that the increase in extracellular adenosine plays an important tissue protective role following acute injury (Volmer et al. 2006; Eckle et al. 2007; Reutershan et al. 2009). These responses include attenuating the inflammatory response by decreasing immune cell recruitment in the local tissue as well as enhancing endothelial barrier function in the lung. Specifically, the adenosine A_2B_ receptor (ADORA2B) dampens inflammation and improves vascular barrier function in acute lung injury models (Schingnitz et al. 2010).

Acute lung injury models involving hypoxic environments for pulmonary cells have demonstrated elevated adenosine levels and protective signaling following acute injury (Koeppen et al. 2011). These include generation of adenosine by CD73 and adenosine signaling through ADORA2B, which provide protective cellular effects (Volmer et al. 2006; Eckle et al. 2007, 2013; Reutershan et al. 2009). However, there are important differences between models of lung injury involving hypoxia and hyperoxia. For instance, in hyperoxic lung injury, different cellular pathways are activated including NF*κ*B as opposed to hypoxia inducible factor (HIF) activation in hypoxia (D'Angio and Finkelstein 2000). Extrapolating research of adenosine's role in hypoxic models of lung injury could lead to incorrect assumptions in hyperoxic conditions, and it is important to determine the role of adenosine in hyperoxic lung injury.

Adenosine signaling has not been extensively studied in hyperoxic lung injury. Thiel et al. used a polymicrobial infection model of ALI in a hyperoxic environment to demonstrate that exposure to hyperoxia exacerbated pulmonary inflammation and edema following an inflammatory insult by dampening the protective role of adenosine (Thiel et al. 2005). Administration of an ADORA2A receptor agonist protected the lung from the inflammatory effect of combined hyperoxia and inflammatory insult. While showing that oxygen supplementation exacerbated acute lung injury by blunting the physiologic protective response elicited by hypoxia including alterations to the adenosine‐mediated tissue protective pathway, they allude to a direct injury by hyperoxic exposure but did not directly test this. Their study does support the concept that hyperoxia represents a different pathology than models of acute lung injury involving local hypoxia and reaffirm the need to evaluate the role of adenosine in hyperoxic lung injury.

In this study, we hypothesized that exposure to a hyperoxic environment causes an inflammatory response in the lung including inflammatory cell recruitment and pulmonary edema that is associated with an increase in extracellular adenosine levels that signal primarily through the ADORA2B receptor to protect lung tissue by attenuating pulmonary inflammation and edema.

## Materials and Methods

### Animal model

Male C57Bl/6 mice, 8–12 weeks old, were ordered from Harlan Laboratories. *CD73*^*−/−*^ mice and *Adora2B*^*−/−*^ mice were generated on C57Bl/6 background and genotyped as previously reported (Volmer et al. 2006; Zhou et al. 2011). Mice were exposed to room air or 95% hyperoxia for 72 h (*n* = 14 for room air wild‐type, *n* = 17 for hyperoxia wild‐type, *n* = 12 for room air *CD73*^*−/−*^, *n* = 12 for hyperoxia *CD73*^*−/−*^, *n* = 13 for room air *Adora2B*^*−/−*^, *n* = 14 for hyperoxia *Adora2B*^*−/−*^). To expose mice to hyperoxic conditions, mice were placed in an A‐Chamber (Biospherix, Lacona, NY) equipped with ProOx 110 controller (Biospherix) in the same room as room air control animals. Oxygen concentrations were monitored continuously and maintained at 95 ± 2% in the chamber at all times. The room was regulated with a 12 h light‐dark cycle. Food and water supplied ad lib. Maintenance and care of animals were in accordance with guidelines set by the Animal Welfare Committee at the University of Texas Health Science Center at Houston and all experiments were approved by the University Of Texas Health Science Center at Houston Animal Welfare Committee.

### Plasma, bronchoalveolar lavage fluid, and histology

Bronchoalveolar lavage fluid (BALF) was obtained as described previously (Wakamiya et al. 1995). Briefly, at time of sample collection, mice were anesthetized with avertin and blood was collected and centrifuged to isolate plasma. Lungs were lavaged 4 times with 0.3 mL PBS containing 10 *μ*mol/L dipyridamole (Sigma–Aldrich, St. Louis, MO), 10 *μ*mol/L ADA‐inhibitor deoxycoformycin (dCF; R&D Systems Inc, Minneapolis, MN), and 10 *μ*mol/L *αβ*‐methylene ADP, which pooled approximately 1.0 mL fluid. A sample was separated for cell count determination using a hemocytometer. The remaining BALF was centrifuged and supernatant and cell pellet were stored for further analyses. Protein concentration in BALF was determined using Bio‐Rad protein assay (Bio‐Rad, Hercules, CA) with BSA standards. After lavage, the pulmonary vasculature was perfused with PBS, lungs were inflated with constant pressure of 20 cm H_2_O and fixed in formalin for 24 h. Lungs were processed and paraffin embedded. Five micrometer sections were cut and used for histology and immunohistochemistry.

### Cytokine analysis

A cytokine ultrasensitive immunoassay (Meso‐Scale Discovery, Gaithersburg, MD) was used to detect the concentrations of CXCL1 and IL‐6 in BAL fluid collected from room air and hyperoxia exposed mice. The plate was read on the MSD detector (Sector Imager 2400; MSD, Gaithersburg, MD). Standard curves were drawn by using the manufacturer's software and cytokine concentrations were determined by interpolation.

### Adenosine measurement

Adenosine concentrations in BALF supernatant were measured using HPLC. One hundred microliters of BALF supernatant was loaded in the HPLC meter per reading and the flow rate was set at 1.5 mL/min. The representative peaks were identified and quantitated by running known external standard curves. Urea was measured from plasma and BALF using Urea Measurement Kit (QuantiChrom Urea Assay Kit; Bioassay Systems, Hayward, CA) and alveolar lining concentration of adenosine was calculated using ratio of concentration of BALF compared to plasma as described previuosly (Rennard et al. 1986).

### Histology and immunohistochemistry

Mouse lungs were collected and fixed in 10% formaldehyde for at least 24 h. Lungs were then dehydrated, paraffin embedded, and sectioned (5 *μ*m). Sections were rehydrated and stained with H&E (Sigma–Aldrich) according to manufacturer's instructions. For occludin immunostaining, sections were quenched with 3% hydrogen peroxide, incubated in citric buffer (VectorLabs, Burlingame, CA) for antigen retrieval, and blocked with Avidin/Biotin Blocking System (VectorLabs). Slides were then blocked with 5% normal goat serum and incubated with primary antibody for Occludin (1:100, rabbit polyclonal; Invitrogen, Carlsbad, CA, 4°C overnight). Slides were incubated with appropriate secondary antibody (1:1000, VectorLabs) for 1 h, and ABC Elite streptavidin reagents for 30 min at room temperature. Finally, slides were developed with 3,3‐diaminobenzidine (Sigma–Aldrich) and counterstained with methyl green. Immunohistochemistry was quantitated using ImagePro software (MediaCybernetics, Rockville, MD). At 20× magnification, pulmonary vessels were identified and pixels with occludin staining were identified using airway epithelial signal in the same field as equilibrative factor for occludin signal in the vessel wall. Pixels were counted for each vessel and then normalized for area of the vessel. Five vessels per slide were quantitated and averaged per slide.

### Western blotting

Lungs were pulverized and protein was extracted using RIPA lysis buffer (50 mmol/L Tris‐HCl PH 7.4, 150 mmol/L NaCl, 1% NP‐40) containing a protease inhibitor cocktail (Thermo Fisher Scientific, Fair Lawn, NJ). Equal amounts of protein were separated on SDS‐PAGE and transferred to nitrocellular membranes. The membranes were then blocked with 5% (w/v) nonfat milk, washed with Tris‐buffered saline–Tween‐20 (TBST), and incubated with primary antioccludin antibodies (1:1000 rabbit; Cell‐Signaling Technology, Danvers, MA) or anti‐GAPDH antibodies (1:20,000 mouse monoclonal; Life Technologies, Grand Island, NY) over night at 4°C. Membranes were then rinsed, incubated with corresponding secondary antibodies conjugated to horseradish peroxidase (Jackson ImmunoResearch, West Grove, PA) for 1 h at room temperature, and developed with Pierce ECL Western Blotting Substrate (Thermo Fisher scientific).

### Real‐time PCR

Total RNA was isolated from frozen lung tissue using Trizol reagent (Life Technologies). RNA samples were then DNAse treated (ArticZymes, Tromso, Norway) and subjected to quantitative real‐time RT‐PCR. Specific transcript levels for the mouse CD73, Adora1, Adora2A, and Adora2B were determined by normalization to 18s RNA and are presented as mean normalized transcript levels using the comparative Ct method (2ΔΔCt).

### Statistical analysis

Outliers were identified using ROUT method. *P*‐values were determined using 2‐tailed *t*‐test and one‐way analysis of variance (ANOVA) followed by Tukey multiple comparisons using GraphPad Prism version 6.00 for Windows (GraphPad Software, La Jolla, CA, www.graphpad.com). All results are presented as mean ± SEM. All error bars on graph represent SEM.

## Results

### Adenosine levels are elevated in hyperoxia

**Figure 1.**
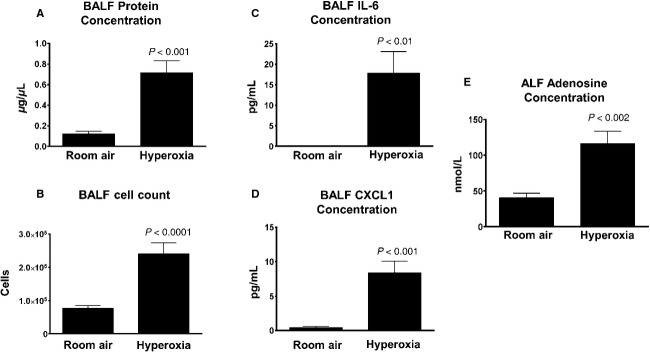
Adenosine levels are elevated in hyperoxic lung injury. Wild‐type mice were exposed to either room air or hyperoxia for 72 h (*n* = 14 for room air, *n* = 17 for hyperoxia). BALF analysis showed increase in protein concentration (A), cell count (B), and cytokines IL‐6 (C) and CXCL1 (D) indicating pulmonary edema and inflammatory cell infiltration. Adenosine concentration in the alveolar lining fluid (ALF) was found to be elevated approximately threefold in mice exposed to hyperoxia (E) (*n* = 10 for room, *n* = 13 for hyperoxia).

We hypothesized that cellular injury caused by exposure to high oxygen concentrations leads to elevated extracellular adenosine levels. To test this hypothesis, we exposed wild‐type C57Bl/6 mice to a 95% oxygen environment or room air for 72 h at which point mice were sacrificed for collection of samples for evaluation of pulmonary inflammation, edema, and adenosine concentration. BALF obtained from C57Bl/6 mice exposed to hyperoxia had increased protein concentrations, cell counts, and IL‐6 and CXCL‐1 concentrations compared with room air (Fig. 1A–D). These elevations demonstrate the inflammation and pulmonary edema that are characteristics of hyperoxic lung injury. We then measured adenosine in BALF from mice exposed to hyperoxia and using urea concentration from BALF and plasma, calculated the alveolar lining fluid concentration (ALFC) of adenosine. We found a threefold increase in adenosine concentration with hyperoxic exposure compared to room air (Fig. 1E). These experiments demonstrate that exposure to a hyperoxic environment is associated with pulmonary inflammation and edema that correspond to a significant increase in extracellular adenosine levels.

### Loss of CD73‐mediated adenosine production worsens hyperoxic lung injury

**Figure 2.**
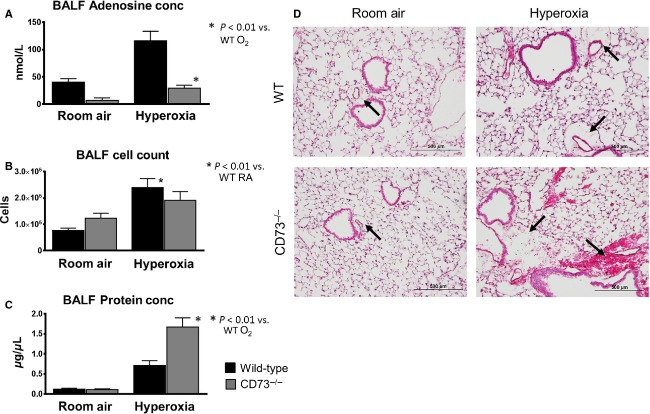
Loss of CD73 adenosine production worsens pulmonary edema in hyperoxic lung injury. *CD73*^*−/−*^ mice were exposed to room air or 95% oxygen environment for 72 h (*n* = 12 for each group). BALF analysis showed decreased adenosine concentrations in *CD73*^*−/−*^ mice in room air and hyperoxia (A). *CD73*^*−/−*^ mice had no change in BALF cell count in hyperoxia (B) but did have an increase in BALF protein concentration (C) indicating worsened pulmonary edema. Histology demonstrated increase in fluid accumulation perivascularly in hyperoxia compared to room air, and exaggerated perivascular fluid in *CD73*^*−/−*^ mice with red blood cell extravasation (black arrows) (D).

To evaluate the role of elevated extracellular adenosine in hyperoxic lung injury, we used *CD73*^*−/−*^ mice to decrease adenosine production and then measured pulmonary inflammation and edema. CD73 is the enzyme that performs the last step of dephosphorylation in the extracellular production of adenosine from ATP. Therefore, *CD73*^*−/−*^ mice are not able to generate extracellular adenosine from ATP that is released during cellular injury. We exposed *CD73*^*−/−*^ mice to 95% oxygen or room air for 72 h. Analysis of BALF confirmed a decrease in ALFC adenosine concentrations in CD73^−/−^ animals exposed to hyperoxia (Fig. 2A). We next analyzed cell counts and protein concentration in BALF to evaluate the effect of decreased extracellular adenosine production on pulmonary edema and inflammation. Protein concentrations in the BALF from *CD73*^*−/−*^ mice in hyperoxia were significantly elevated compared to wild‐type mice in hyperoxia (Fig. 2B). Although cell counts in the BALF from *CD73*^*−/−*^ mice in hyperoxia were elevated from CD73^−/−^ mice in room air, there was no difference in BALF cell counts between CD73^−/−^ mice and wild‐type C57Bl/6 mice following hyperoxic exposure (Fig. 2C).

Lung sections were cut and stained for analysis (Fig. 2D). Wild‐type mice exposed to hyperoxia showed an increase in perivascular fluid accumulation (black arrows). However, *CD73*^*−/−*^ mice in hyperoxia had increased perivascular fluid compared to wild‐type animals in hyperoxia and in some cases red blood cell extravasation. There were no changes between *CD73*^*−/−*^ and wild‐type mice in room air. These histologic findings are consistent with the increase in protein concentration in BALF. Taken together, these findings indicate loss of CD73‐mediated adenosine production in hyperoxia leads to worsened pulmonary edema possibly due to a loss of pulmonary vascular barrier function without an effect on inflammatory cell infiltration in the lung.

### Loss of ADORA2B worsens hyperoxic lung injury

**Figure 3.**
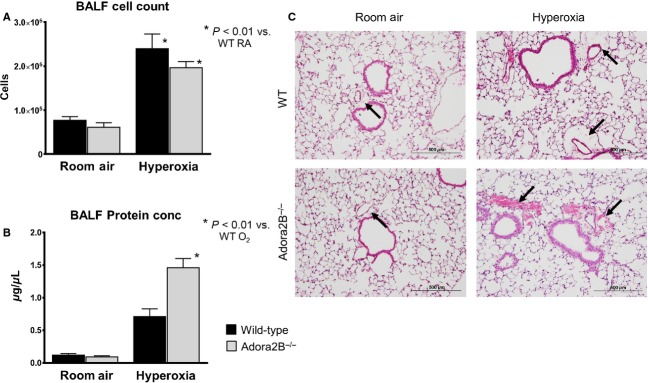
Loss of ADORA2B worsens pulmonary edema in hyperoxic lung injury. *Adora2B*^*−/−*^ mice were exposed to room air or 95% oxygen environment for 72 h (*n* = 13 for room air, *n* = 14 for hyperoxia). *Adora2B*^*−/−*^ mice had no change in BALF cell count in hyperoxia (A) but did have a significant elevation in BALF protein concentration (B) indicating worsened pulmonary edema. H&E staining of representative sections shows an increase in perivascular fluid accumulation in hyperoxia compared to room air that is exaggerated and demonstrates red blood cell extravasation in *Adora2B*^*−/−*^ mice (black arrows) (C). Panels for wild‐type mice are the same as presented in Figure 2.

ADORA2B plays an important role in acute injury response in the lung, specifically to preserve vascular barrier function (Eckle et al. 2008a). We used *Adora2B*^*−/−*^ mice to determine if the protective effect of adenosine was mediated by activation of ADORA2B. We exposed *Adora2B*^*−/−*^ mice to 95% oxygen or room air for 72 h. Adenosine concentrations in BALF were elevated in *Adora2B*^*−/−*^ mice exposed to hyperoxia compared to room air, but there was no difference from wild‐type mice exposed to hyperoxia (data not shown). Protein concentrations in the BALF from *Adora2B*^*−/−*^ mice in hyperoxia were significantly elevated compared to wild‐type mice in hyperoxia similar to *CD73*^*−/−*^ mice (Fig. 3A). Additionally, cell counts in the BALF from *Adora2B*^*−/−*^ mice in hyperoxia were elevated compared to *Adora2B*^*−/−*^ in room air, but were not significantly different from wild‐type mice in hyperoxia (Fig. 3B).

Lung sections from wild‐type and *Adora2B*^*−/−*^ mice were cut and stained for analysis (Fig. 3C). *Adora2B*^*−/−*^ mice exposed to hyperoxia had increased perivascular fluid compared to wild‐type animals in hyperoxia and in some cases red blood cell extravasation similar to hyperoxia exposed *CD73^−/−^* mice. There was no difference between *Adora2B*^*−/−*^ and wild‐type mice in room air. These histologic findings are consistent with the increase in protein concentration in BALF, and the results from BALF analysis and histology are consistent with the results from *CD73*^*−/−*^ mice. These findings suggest that adenosine signals through the ADORA2B receptor to preserve pulmonary vascular barrier function in hyperoxic lung injury.

### Occludin is decreased by hyperoxia, an effect exacerbated by loss of CD73 and ADORA2B

**Figure 4.**
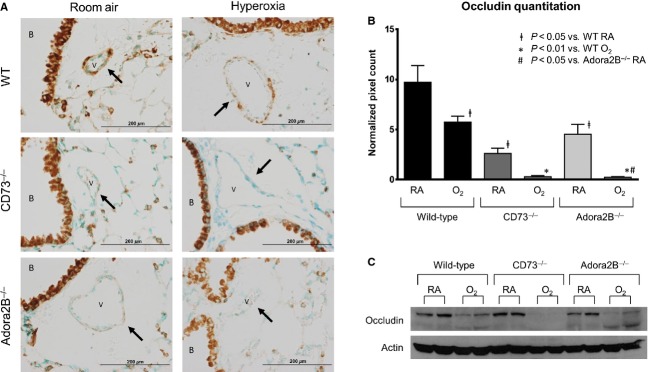
Occludin is decreased in pulmonary endothelial cells of *CD73*^*−/−*^ and *Adora2B*^*−/−*^ mice with hyperoxic exposure. Representative immunohistochemistry staining for occludin (A), a cellular adhesion protein important in pulmonary vascular barrier function, in wild‐type, *CD73*^*−/−*^ and *Adora2B*^*−/−*^ mice in room air and hyperoxia. Occludin was decreased in pulmonary vasculature in wild‐type animals exposed to hyperoxia, and decreased in *CD73*^*−/−*^ and *Adora2B*^*−/−*^ mice in room air. However, occludin was nearly nonexistent in CD73^−/−^ and Adora2B^−/−^ mice exposed to hyperoxia (black arrows). Occludin‐staining quantitation demonstrates these findings (B). Western analysis of whole lung lysates for occludin demonstrates decreased occludin in hyperoxia and near loss of occludin in *CD73*^*−/−*^ and *Adora2B*^*−/−*^ mice in hyperoxia (C).

Cellular adhesion proteins are critical components in pulmonary vascular barrier function. Occludin is one of the cellular adhesion proteins that play an important role in barrier function in the lung (McCarthy et al. 1996; You et al. 2012). Additionally, occludin can be regulated by calpain (Chun and Prince 2009) and MMP‐2 (Liu et al. 2012) which can be regulated by the downstream adenosine pathway component cAMP (Peracchia et al. 1997; Shiraha et al. 2002). We, therefore, evaluated if regulation of occludin was affected by adenosine signaling. Lung sections from C57Bl/6, *CD73*^*−/−*^ and *Adora2B*^*−/−*^ mice in both room air and hyperoxia were subjected to immunohistochemistry using an occludin antibody (Fig. 4A). Wild‐type animals in room air had strong occludin signal in the arteriole walls, and this staining was decreased in wild‐type mice exposed to hyperoxia. In room air, both *CD73*^*−/−*^ and *Adora2B*^*−/−*^ mice had decreased amounts of occludin signal in the pulmonary arteriole. Moreover, when *CD73*^*−/−*^ and *Adora2B*^*−/−*^ mice were exposed to hyperoxia, almost all occludin signal was lost in pulmonary arterioles. We quantified occludin immunohistochemistry and found a significant decrease in occludin signal in *CD73*^*−/−*^ and *Adora2B*^*−/−*^ mice in room air, and near complete loss of signal in knockout mice exposed to hyperoxia (Fig. 4B).

We also measured occludin protein in whole lung lysates from wild‐type, *CD73*^*−/−*^ and *Adora2B*^*−/−*^ mice in room air and hyperoxia (Fig. 4C). Western blot showed a decrease in occludin protein in wild‐type animals in hyperoxia compared to room air. There was a significant decrease in occludin levels in *CD73*^*−/−*^ and *Adora2B*^*−/−*^ mice in hyperoxia. The wild‐type and *Adora2B*^*−/−*^ mice in hyperoxia also had increased signal of the occludin degradation products represented by the lower band on the western blot. Interestingly, *CD73*^*−/−*^ mice did not have evidence of occludin or breakdown products.

These results demonstrate that hyperoxia causes a decrease in occludin, a cellular adhesion molecule important for pulmonary vascular barrier function. This decrease is exaggerated with loss of extracellular adenosine and ADORA2B signaling suggesting that adenosine promotes vascular barrier function in hyperoxia through protection of occludin levels in the lung.

### *CD73* and *ADORA2B* expression are increased in hyperoxic lung injury

**Figure 5.**
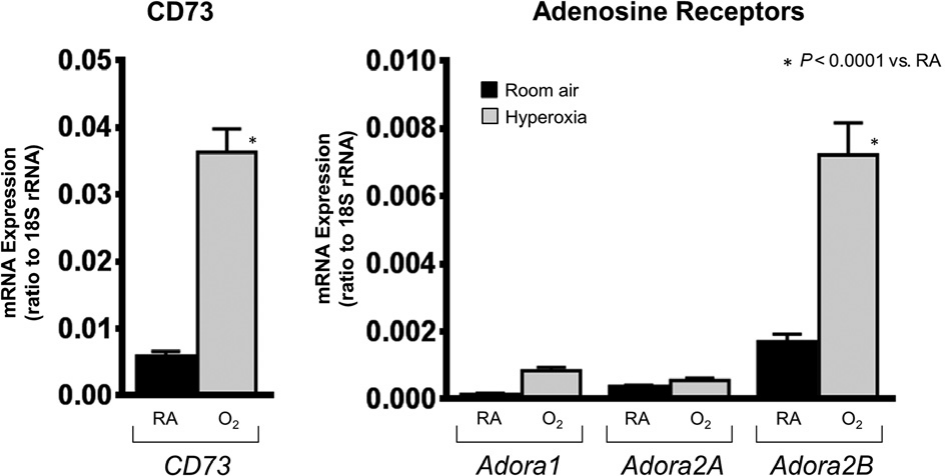
CD73 and Adora2B receptor are upregulated in hyperoxic lung injury. Quantitative PCR analysis of whole lung lysate showed increased expression of *CD73* and *Adora2B* in mice exposed to hyperoxia compared to room air. (*n* = 6 for room air, *n* = 5 for hyperoxia).

*CD73* and *Adora2B* expression are upregulated following models of acute lung injury (Kong et al. 2006; Eckle et al. 2014), and this up‐regulation is due to HIF‐1*α* (Synnestvedt et al. 2002; Kong et al. 2006; Eckle et al. 2014). Hyperoxic lung injury represents a unique physiologic stress on the cells with activation of different transcription factors (D'Angio and Finkelstein 2000). In fact, HIF‐1*α* is downregulated with hyperoxia exposure (Popescu et al. 2013), and so it was important to evaluate transcript levels of adenosine receptors in this model of lung injury. We confirmed that both *CD73* and *Adora2B* transcript levels were increased in hyperoxic lung injury (Fig. 5). These results are interesting as they are similar to hypoxic lung injury models despite the lack of HIF‐1*α* activation in hyperoxia.

## Discussion

Adenosine has been shown to play an important role in modulating the inflammatory process in many models of lung injury (Eckle et al. 2008a,b; Schingnitz et al. 2010; Karmouty‐Quintana et al. 2013). However, most of these models and processes involve local hypoxic conditions that have been well established to alter expression of adenosine pathway components leading to increases in extracellular adenosine concentrations and enhancement of adenosine signaling. Hyperoxic lung injury represents a different physiology with activation of alternative cellular pathways, yet due to the common use of oxygen in the clinical arena, it is important to understand the role of adenosine in hyperoxic lung injury to identify targets for novel therapies to prevent and treat hyperoxic lung injury. In the current study, we aimed to assess if hyperoxia produces an increase in extracellular adenosine levels, and if elevated, whether adenosine contributes to attenuation of inflammation and lung protective responses. We found that exposure to high levels of oxygen leads to elevations of extracellular adenosine concentrations. These substantial elevations are not surprising, given our understanding of the mechanism of lung injury by hyperoxia. Moreover, the loss of adenosine production or signaling resulted in enhanced disease, suggesting that the adenosine produced in hyperoxic injury has a protective role.

Oxygen exposure in the lung produces ROS through multiple mechanisms including oxidative phosphorylation in the mitochondria (Kallet and Matthay 2013). ROS production increases linearly with intracellular P_O2_, and at high levels of supplemental oxygen, cellular antioxidant defensive mechanisms such as superoxide dismutase are overwhelmed. Free ROS capture electrons from cellular components which can damage cell membranes and DNA, alter enzymatic function and potentially lead to cell death (Kallet and Matthay 2013). This cellular damage starts an inflammatory cascade during which immune cells are recruited to the area and are activated, consequently producing additional ROS that contribute further to local cellular damage. This combination of cellular damage, inflammation, and cell death will produce an efflux of ATP out of cells in the area of injury, and through dephosphorylation by ectonucleotidases, would lead to the elevations of extracellular adenosine that we found in our study. This is supported in our study by the lower extracellular adenosine concentrations in *CD73*^*−/−*^ mice.

A major objective of this study was to determine the role of elevated levels of adenosine in the modulation of inflammation in hyperoxic lung injury. Elevated adenosine levels have been found to attenuate lung inflammation and pulmonary edema in models of lung injury involving tissue hypoxia (Volmer et al. 2006; Eckle et al. 2007; Reutershan et al. 2009). However, there are important differences in cellular responses in hyperoxic and hypoxic environments. Although we expected a similar role for adenosine, it was important to clarify adenosine's role in hyperoxic lung injury. Hypoxia stabilizes molecules such as HIF‐1*α* that alters expression of adenosine pathway components including upregulation of CD73 and Adora2B (Synnestvedt et al. 2002; Kong et al. 2006; Hart et al. 2011; Eckle et al. 2014). The combined effect is enhancement of adenosine signaling in order to protect the lung from tissue damage with increased production of adenosine. Additionally, upregulation of the ADORA2B receptor has been implicated in adenosine's protection of vascular barrier function in hypoxic lung injury (Schingnitz et al. 2010; Eckle et al. 2014). In hyperoxic lung injury, HIF‐1*α* is not stabilized (Popescu et al. 2013) but ROS activate redox‐sensitive transcription factors and affect protein kinase activity to modulate stress response and apoptotic pathways including NF‐*κβ* (Michiels et al. 2002), NRF2 (Cho et al. 2002), STAT (Lee et al. 2000), and the MEK pathway (Jones and Agani 2003).

Despite the differences between hypoxia and hyperoxia, given the levels of extracellular adenosine, we hypothesized that the elevation in extracellular adenosine in hyperoxic lung injury plays a similar role to attenuate the inflammatory response. Using *CD73*^*−/−*^ mice to decrease extracellular adenosine production, our results demonstrate that reducing adenosine signaling worsens pulmonary edema without affecting inflammatory cell recruitment to the lung. Adenosine enhances endothelial barrier function in vitro (Lu et al. 2010), and elevated adenosine levels attenuate pulmonary edema in inflammatory models of ALI (Lu et al. 2010). Our results demonstrating that elevated adenosine levels promote pulmonary barrier function in hyperoxic lung injury are consistent with these studies.

However, the lack of attenuation on inflammatory cell recruitment by adenosine in hyperoxic lung injury is surprising given the results in other models of lung injury. Studies using *CD39‐* and *CD73*‐deficient mice and inhibitors showed exacerbation of tissue inflammation and injury in models of ischemia and hypoxia (Volmer et al. 2006; Eckle et al. 2007; Reutershan et al. 2009). In these studies, blocking elevations of adenosine following lung injury led to an increase in inflammatory cell recruitment to the lung, which is not consistent with our findings. The difference may be due to compensatory pathways activated in hyperoxia versus hypoxia. For instance, Thiel et al. showed that hyperoxia affects the physiological response to injury and nullifies much of the signaling caused by hypoxic conditions, including blunting the increased expression of *Adora1* and *Adora2A* under hypoxic conditions (Thiel et al. 2005). This highlights the importance of the current study evaluating adenosine signaling in hyperoxia.

We focused our attention on the ADORA2B receptor since studies in acute lung injury in the lung have found that this receptor plays a major role in the preservation of barrier function signaling of adenosine following injury (Eckle et al. 2008a,b; Zhou et al. 2011). Similar to our results with CD73 knockouts, mice lacking ADORA2B had no change in inflammatory cell counts in the lung but a significant increase in protein concentration in BAL fluid when exposed to a hyperoxic environment. This suggests that the ADORA2B receptor plays an important role in adenosine's signaling to protect barrier function in the lung and is consistent with previous work characterizing specific function of the ADORA2B receptor. This is a significant finding as identification of this role for ADORA2B allows for the possibility of targeted therapy that enhances ADORA2B signaling to protect patients on supplemental oxygen from hyperoxic lung injury.

We also attempted to identify the downstream target important for barrier function that appears to be affected by adenosine signaling through the ADORA2B receptor. Occludin is an important protein involved in cellular adhesion (McCarthy et al. 1996; You et al. 2012), and has been found to be modulated by various cellular signaling (Chun and Prince 2009; Liu et al. 2012). We demonstrated using immunohistochemistry that occludin is decreased in the pulmonary vasculature in mice exposed to hyperoxia and essentially absent in pulmonary vasculature in *CD73*‐ and *Adora2B*‐deficient mice. In addition, the western blot analysis of whole lung lysate correlates with decreased occludin signal in hyperoxia and near loss of signal in *CD73*^*−/−*^ and *Adora2B*^*−/−*^ mice exposed to hyperoxia. Given that there is still substantial signal in the epithelium of large airways on immunohistochemistry, this suggests there is a substantial loss of occludin in other parts of the lung as well, including the alveolar epithelium and alveolar capillaries. Taken together, these results suggest that adenosine signaling protects vascular barrier function by preserving endothelial cellular adhesion proteins such as occludin.

Research on cellular regulation of occludin has identified potential pathways by which ADORA2B may be regulating occludin in pulmonary vasculature. Adenosine receptors are G‐protein coupled and alter adenylate cyclase activity. The ADORA2B is G_s_ coupled and activates adenylate cyclase to increase intracellular cAMP (Zhou et al. 2009). cAMP can alter calpain activity (Shiraha et al. 2002) and MMP‐2 expression (Peracchia et al. 1997), both of which can regulate occludin (Chun and Prince 2009; Liu et al. 2012) to decrease occludin levels and weaken barrier function. These signaling pathways provide a possible explanation of how elevations of adenosine could signal through the ADORA2B receptor to regulate occludin.

Degradation of occludin is supported in part by our Western analysis. Evaluation of occludin in whole lung lysate showed a decrease in occludin signal in wild‐type mice in hyperoxia, and further decreased in *CD73‐* and *Adora2B‐*deficient mice in hyperoxia. The degradation products of occludin represented by the lower band on the western were elevated in wild‐type and *Adora2B‐*deficient mice in hyperoxia indicating increased breakdown of occludin. This could represent an increase in occludin degradation by enzymes such as calpain. However, the degradation products were absent in the *CD73*‐deficient mice. This surprising result could be due to additional breakdown in the occludin protein, or it could indicate an alternative regulation of occludin leading to a decrease in total amount of occludin present. This finding suggests the other adenosine receptors may be playing a role as well in the regulation of barrier function by adenosine in hyperoxic lung injury.

Our data support an important role of adenosine in protecting barrier function in hyperoxic lung injury through the ADORA2B receptor. This raises the potential for therapeutic intervention to protect patients from the pulmonary edema seen with hyperoxic exposure. In hypoxic models of lung disease, blockade of adenosine uptake by equilibrative nucleoside transporters (ENTs) to increase the extracellular concentration of adenosine and enhance signaling has also been attempted (Zimmerman et al. 2013). However, adenosine can vasodilate the pulmonary vasculature through the ADORA2A receptor (Pearl 1994) and in combination with oxygen's vasodilatory effect, could actually worsen pulmonary edema. Since this study demonstrates ADORA2B signaling specifically protects vascular barrier function, direct activation of ADORA2B could be exploited therapeutically (Eckle et al. 2008b; Schingnitz et al. 2010). Additionally, Nowak‐Machen et al. have promising results supporting therapeutics aimed at increasing extracellular ATP (Nowak‐Machen et al. 2013), but our results showing adenosine's protective role raises the potential for additive or synergistic therapy, whereby drugs that increase extracellular ATP are used in conjunction with ADORA2B receptor agonists to provide enhanced therapy to prevent hyperoxic lung injury. Additionally, targeting the downstream signaling of adenosine could potentially offer synergistic effects. For instance, calpain inhibitors have been shown to inhibit cellular adhesion protein cleavage in vitro and in vivo (Chun and Prince 2009), and calpain inhibitors have been identified as potential therapeutics for other diseases including Alzheimer's disease (Medeiros et al. 2012), myocardial ischemia/reperfusion injury (Inserte et al. 2012), cataracts (Morton et al. 2013), and diabetic retinopathy (Shanab et al. 2012).

This study also has clinical implications on the use of caffeine in neonatal patients. Caffeine, a nonspecific adenosine receptor antagonist, is widely used in premature neonates for treatment of apnea of prematurity. The Caffeine for Apnea of Prematurity Trial Group found caffeine decreased the risk of BPD in premature infants (Schmidt et al. 2006). Many premature infants are treated with caffeine for apnea of prematurity and BPD prevention for weeks and months as they grow and mature. Although caffeine may be beneficial in the long‐term care of premature neonates, adenosine's protective role in acute lung injury and high levels of oxygen supplementation may be blunted by caffeine. Thus, a neonate on caffeine treatment who has an acute decompensation with an increase in supplemental oxygen may not benefit from adenosine's protective effect in hyperoxic lung injury.

In summary, we have demonstrated that exposure to a hyperoxic environment leads to an increase in extracellular adenosine that acts to preserve pulmonary barrier function through the ADORA2B receptor by modulating the cellular adhesion protein occludin. These results have implications for potential therapies for hyperoxic lung injury, as well as implications for the use of caffeine in neonates.

## Acknowledgments

The authors acknowledge S. E. Welty for his assistance on the hyperoxia model. We also thank W. Jiang for his help with learning the mouse hyperoxia model.

## Conflict of Interest

We have nothing to disclose.
